# Priorities for prehabilitation for patients with upper gastrointestinal cancer: a nominal group consensus study

**DOI:** 10.1007/s00520-025-09844-5

**Published:** 2025-08-18

**Authors:** Robyn J. Stiger, Mark A. Williams, Johnny Collett

**Affiliations:** 1 Oxford Institute of Applied Health Research, Oxford Brookes University, Oxford, UK; 2 Oxford Allied Health Professions Research and Innovation Unit, Oxford University Hospitals, NHS Foundation Trust, Oxford, UK

**Keywords:** Prehabilitation, Upper gastrointestinal cancer, Exercise oncology, Cancer rehabilitation, Nominal group technique

## Abstract

**Purpose:**

Prehabilitation is a broad and evolving concept that is recommended to improve patient outcomes but is not offered routinely to patients undergoing surgery in centres across the UK. The purpose of this study was to combine existing evidence with expert opinion to establish consensus on the key priorities for prehabilitation interventions in patients undergoing surgery for upper gastrointestinal cancer.

**Methods:**

A modified nominal group technique was utilised. Prior to an online meeting, individual ideas were generated in response to questions across three themes: prehabilitation interventions, clinical implementation and behaviour change support, and outcomes and cost-effectiveness. The online meeting included facilitated discussions to clarify and regroup ideas, followed by anonymous ranking of individual ideas. GroupMap™ software was used to collect and analyse the data.

**Results:**

Eight individuals attended the meeting in November 2023, including physiotherapists and dietitians experienced in prehabilitation and an experienced patient advocate. A high level of consensus was achieved across many aspects of prehabilitation, with eight out of 89 ideas achieving 100% consensus agreement (9%) and 31 out of 89 ideas achieving consensus of 70% or higher (35%). The main findings demonstrated complete consensus that prehabilitation should be delivered as a multimodal, multidisciplinary intervention that combines all three core aspects (exercise, nutrition and psychological support).

**Conclusion:**

The study findings are consistent with developing evidence trends and confirmed which core aspects should be incorporated into prehabilitation interventions for patients undergoing upper gastrointestinal surgery. These insights will help to develop prehabilitation services that are integrated across professions and systems for best patient care.

**Supplementary Information:**

The online version contains supplementary material available at 10.1007/s00520-025-09844-5.

## Introduction

Although the rates of cancer diagnosis worldwide are increasing, the number of patients surviving and living with cancer continues to steadily improve [1, 2]. Despite advances in care, the burden of post-surgical complications in patients diagnosed with upper gastrointestinal (GI) cancer remains high, compared with other surgical specialities [3, 4]. Surgery remains the only potential cure; therefore, minimising complications and optimising wellbeing outcomes is a high priority [5–7]. Prehabilitation, which is a broad concept that aims to reduce surgery-related morbidity through modification of risk factors, forms the initial part of the rehabilitation continuum and shifts some of the rehabilitation process to the period before surgery [8, 9]. The main components include physical activity and exercise, nutrition and psychological support and behavioural change [10].

Our recent systematic review [11] demonstrated that whilst there was substantial variation in the interventions evaluated and the outcome measures utilised, prehabilitation interventions appear safe and most effective when delivered as a multimodal, multidisciplinary approach. However, prehabilitation for patients undergoing upper GI surgery is not currently standard practice with no specific clinical guidelines. Thus, there is a need to identify components and mode of delivery of interventions to inform the clinical implementation of upper GI prehabilitation. Co-creation with stakeholders is important to maximise the potential of developing interventions that are likely to achieve changes in practice and have positive impacts on health [12].

The aim of this study was to use a modified nominal group technique process with a mixed key stakeholder group to consider multiple sources of evidence and gain consensus on prehabilitation interventions for patients undergoing upper GI surgery for cancer in the UK. A secondary aim was to gain consensus on service organisation and clinical and cost-effectiveness measures that should be utilised to implement and evaluate prehabilitation interventions.

## Methods

Prehabilitation is an evolving concept with complexities and uncertainties; therefore, a consensus method study approach was utilised to combine current best evidence with key stakeholder opinions. An online modified nominal group technique (mNGT) approach was deemed most appropriate, allowing for geographical inclusivity of key stakeholders and making best use of their time [13–18]. This method enables generation and exchange of ideas and opinions within a staged, facilitated process.

The study is reported according to ACcurate COnsensus Reporting Document (ACCORD) [19] (Supplementary file 1).

### Recruitment

Given the multimodal complexity of included interventions, participants were recruited from a range of key stakeholder backgrounds, including upper GI patient representatives, clinicians and health-care researchers. The target sample size was 15 to 30 participants, allowing for up to three groups of around five people [20]. The study was advertised by cancer and prehabilitation professional networks in the UK (“Physios in Exercise Oncology” and the NHS England “Prehabilitation Steering Group”). It was also shared on the social media channels X (formerly Twitter) and LinkedIn. Potential participants volunteered by completing an expression of interest form via a Google form embedded within an information slide.

### Selection and consenting

To be eligible, clinicians were required to have experience of working in prehabilitation, exercise rehabilitation and/or with patients diagnosed with cancer. Researchers were required to have experience of research within the areas of surgery or oncology. Patients or carers were required to have personal experience with upper GI cancer but not actively undergoing chemotherapy, radiotherapy or awaiting surgical intervention. Following screening of the expression of interest form responses, eligible participants were sent the proposed date of the online meeting, the study information sheet and the consent forms to complete and return via email.

### Stages of the mNGT process

**Fig. 1:**
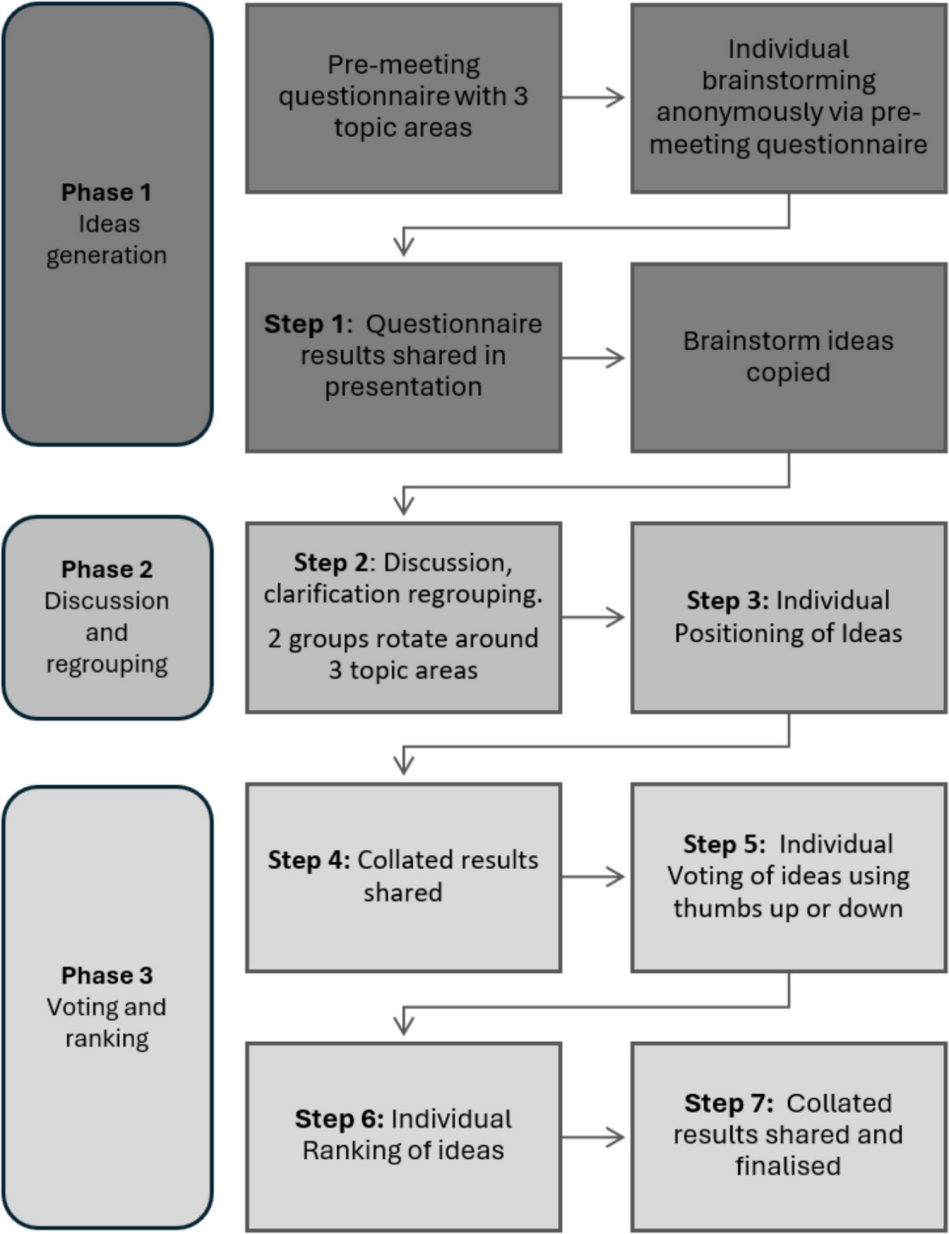
Flow diagram of modified Nominal Group Technique (mNGT) process

A team of four facilitators coordinated the online mNGT process. An overview of their roles is given in Supplementary file 2. The mNGT process was conducted in three phases (Fig. 1). The individual ideas generation (phase one) was completed *before* the face-to-face meeting via a pre-meeting questionnaire. The collated results of this questionnaire were then fed back to the group in the early part of the online meeting. This was followed by facilitated discussions to clarify and regroup ideas and ensure participant understanding (phase two). The final part of the process was individual voting and ranking of ideas (phase three) [13–16]. All stages were facilitated using GroupMap™ software to collect, collate, analyse and present the anonymous data. All maps were set to ensure participant anonymity with no answers attributable to individuals at any point in the study process.

### Completion of the pre-meeting questionnaire

**Fig. 2:**
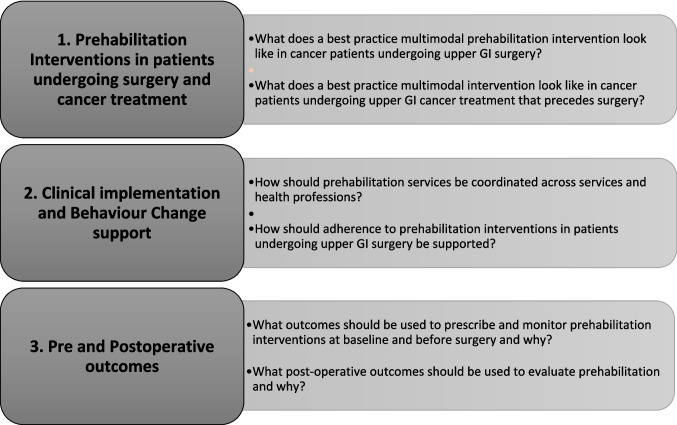
Questions asked for the three topic areas in the pre-meeting questionnaire

The pre-meeting questionnaire administered via GroupMap™ consisted of three key sections with two questions for each section. The themes of these sections were prehabilitation interventions; clinical implementation and behaviour change support; outcomes and cost-effectiveness (Fig. 2). The participants were asked to individually brainstorm and input as many free-text ideas as they wished in response to the six questions. Participants could return to the questionnaire multiple times, amending or adding to their answers until the day before the consensus meeting, at which point the questionnaire was closed for collation by the research team. The map settings ensured that participants could only view their own answers at this stage of the process and not those of other participants.

### Online consensus meeting process

A one-day meeting was held online via Zoom™. The meeting included ice-breaker activities to build group cohesion and psychological safety [21], as well as an overview of the agenda and expectations for the day (Supplementary file 2).

The online meeting mNGT process followed seven steps (Fig. 1). A summary of the pre-meeting information was presented to the participants, followed by sharing the results of the initial ideas generated by the pre-meeting questionnaire (step 1). In two groups, the participants took part in facilitator-led discussions in breakout rooms where they clarified and regrouped the ideas (step 2). This enabled refinement of the ideas prior to the ranking process. Both groups rotated through the three topic areas, building on the responses from the initial questionnaire, as well as on the work of the previous group where appropriate (Supplementary file 2). During this phase, only the facilitators were responsible for editing the ideas according to the group discussions on the relevant GroupMap™. Next, using GroupMap™ software, individual positioning, voting and ranking processes for each of the three topic areas was conducted by the participants, with the whole group working together (steps 3 to 7). Preliminary individual positioning of ideas was performed (step 3). The results were immediately shared by the facilitator and discussed to ensure agreement (step 4). The participants then voted on the ideas using a thumbs up/thumbs down function in GroupMap™ (step 5). Once complete, the facilitators shared the results with the participants who then individually ranked the ideas in their order of preference (step 6). Finally, the collated group results of the positioning, voting and ranking processes were shared with the participants, with invited comments and discussion prior to being finalised (step 7). Once the process (steps 3 to 7) had been followed for all three topic areas, the online meeting was officially closed, and the participants were thanked for their time. Copies of the results for each of the three maps were emailed to the participants the following day.

### Analysis

Ideas were combined where overlapping themes were identified; these were discussed and regrouped and the frequency data from individual positioning, voting and ranking collated in an overall map for each topic. The maps were downloaded from GroupMap™. With no universally accepted level of consensus [22], we chose to pre-specify that ideas with at least 70% agreement (5 out of 7 participants) were considered to have achieved consensus. This aligns with recommendations by Murphy et al. [13] and frequent use of this threshold in similar studies [23].

## Results

### Participant recruitment and characteristics

The expression of interest form was completed by 27 individuals who all met the eligibility criteria. Eleven returned the consent form and were sent the anonymous pre-meeting questionnaire: physiotherapists (*n* = 6), dieticians (*n* = 2), surgeon (*n* = 1), clinical exercise physiologist (*n* = 1) and upper GI cancer survivor (*n* = 1). Seven participants responded anonymously to the pre-meeting questionnaire, contributing 59 initial ideas across the three topic areas. Eight participants attended the online consensus meeting in November 2023: physiotherapists (*n* = 4), dieticians (*n* = 2), clinical exercise physiologist (*n* = 1), patient representative and survivor of upper GI cancer (*n* = 1). Their demographic details, professional background and clinical and research experience are summarised in Table 1.

**Table 1:** Summary of participant characteristics and area and level of expertise

No	Gender	Area of expertise	Experience working with patients diagnosed with cancer	Years working in research	UK Region/Location
1	Female	Physiotherapist working in intensive care and surgery with patients undergoing upper GI surgery	> 5 years	N/A	South East
2	Female	Physiotherapist working in upper GI prehabilitation	> 5 years	N/A	North East
3	Male	Physiotherapist working clinically in upper GI prehabilitation	> 1 year but < 5 years	N/A	South East
4	Female	Physiotherapist and PhD candidate researching prehabilitation for patients with lung cancer	> 1 year but < 5 years	> 1 year	West Midlands/Jordan
5	Female	Dietician working clinically in lung and colorectal prehabilitation. PhD candidate researching nutritional oncology	> 5 years	> 1 year	South East
6	Female	Dietician working clinically in prehabilitation	> 5 years	N/A	Scotland
7	Male	Clinical Exercise Physiologist — expert in cardiac rehabilitation but also has experience of working with people with cancer	> 5 years	> 5 years	South East
8	Male	Patient advocate. OG cancer survivor (over 10 years). Brother recently diagnosed with upper GI cancer but did not survive. Works with research groups as a patient advocate, e.g. National Cancer Research Institute (NCRI) and National Institute for Health and Care Research (NIHR)			South East

### Findings from the online consensus panel meeting

Following the discussion and clarification stage (step 2), 89 ideas across the three topics areas were taken forward, with the number of ideas for each topic increasing: prehabilitation interventions (*n* = 28), implementation and behaviour change support (*n* = 31) and outcomes (*n* = 30). Seven participants completed the final voting and ranking processes as one had to return to their clinical role unexpectedly after the morning session.

Overall, eight out of 89 ideas (9%) achieved 100% consensus agreement and 31 out of 89 ideas (35%) achieved consensus of over 70% across the three themes. These main findings are summarised in Table 2 according to the level of agreement.

**Table 2:** Summarised ideas achieving consensus

Theme	Ideas	% level of agreement
Interventions	• Multimodal intervention — physiotherapy, dietetics and psychology • Individualised specialist dietetic support	**100**
Implementation and behaviour change	• Tailored to the individual • Ensure the patient opinion is valued, i.e. what do they want to achieve out of prehab • Peer support — hearing from those who have gone through the programme previously • Involvement of family or carers	
Outcomes	• Patient stories — qualitative data • Mental health — anxiety, stress, loss	
Interventions	• Integration of peer support groups into multimodal prehabilitation interventions • Education on what to expect post-op and on the expected progression of activity in hospital and on discharge	**86**
Implementation and behaviour change	• Agreed level of training for anyone delivering aspects of programme/intervention appropriate level of staff • Goal setting • Helping people to understand why each component is suggested • Adequate education to patients of prehab importance and reasons for each change • Family education, support groups and group sessions to support adherence. Along with the continuous support from health professions in different stages of the patient journey	
Outcomes	• Body composition at baseline, pre-surgery and discharge post-surgery with/without rehabilitation • Grip strength — gross upper limb strength test — baseline, 6–8 weekly, pre-surgery, post-surgery and post-NACT • 30-s sit to stand — gross lower limb strength and endurance test — could be done face-to-face or via video call if needed	
Interventions	• Early access to MDT led course/programme of nutrition education/physical activity/psychological support/lifestyle coaching, e.g. 6-week rolling programme of support with access to specialists for those who need more tailored advice, e.g. specialist dietitians • Exercise prescription tailored to the individual patient adhering to SAID principles • Integration of smoking cessation and life-style changes through dedicated support • Single point of contact person — coordinating role	**71**
Implementation and behaviour change	• Monthly/quarterly meeting for prehab team leads — coordination • Support for patients could be virtual video calls or via an app, but with an option for face to face for those unable to use the technology	
Outcomes	• Functional capacity, i.e. CPET, 6MWD or ISWT • Nutritional status • Weight and *BMI* • Fatigue • Function • Quality of life — (i.e. EQ-5D pre and post op, as generic measure of quality-of-life pre-op vs. post-op vs. 6–12 m quality of life, EORTC-QLQ-30 disease specific providing sub domains including fatigue) • Cost-effectiveness — length of stay (ICU, HDU, Hospital), medication usage and EQ-5D)	

*6MWD* six-minute walk test/distance, *BMI* body mass index; *CPET* cardiopulmonary exercise testing, *EORTC QLQ-30* European Organisation for Research and Treatment of Cancer 30-item, *EQ-5D-5L* 5 level EQ-5D version measuring health related quality of life, *HDU* high dependency unit, *ICU* intensive care unit, *ISWT* incremental shuttle walk test, *MDT* multidisciplinary team, *NACT* neoadjuvant chemotherapy, SAID principles specific adaptations to imposed demands

### Prehabilitation interventions

Twenty-eight ideas were generated in response to the questions regarding prehabilitation interventions, with eight achieving a consensus agreement of over 70%. The results of the individual positioning, thumbs up/down-voting and final individual ranking of the ideas and levels of agreement downloaded from GroupMap™ for prehabilitation interventions are displayed in Supplementary file 3.

There was 100% consensus agreement that prehabilitation should be a multimodal, multidisciplinary intervention involving physiotherapy, dietetics and psychological input, with further detailed description regarding the multimodal interventions reported. There was also 100% agreement that the specialist dietetic support should be individualized. There was 86% consensus agreement on the need for peer support groups to be integrated into the multimodal prehabilitation intervention, as well as the inclusion of patient education addressing postoperative expectations and awareness. There was 71% consensus agreement regarding early access to multidisciplinary led prehabilitation courses, as well as the exercise aspect being guided by SAID (Specific Adaptations to Imposed Demands) principles to ensure progressive overloading to maximise training benefits. There was also agreement about the need for smoking cessation and lifestyle change support, as well as access to a prehabilitation coordinator.

### Implementation and behaviour change

Thirty-one ideas were generated in response to the questions regarding the clinical implementation and behaviour change element of prehabilitation with 11 achieving a consensus agreement of over 70%. The results of the individual positioning, thumbs up/down-voting and final individual ranking of the ideas and levels of agreement downloaded from GroupMap™ for prehabilitation implementation and behaviour change are displayed in Supplementary file 3.

There was 100% consensus agreement that prehabilitation should be tailored to the individual and that the patient’s opinion should be valued in terms of what they want to achieve from engaging with prehabilitation. Peer support from those who have previously gone through the programme and involvement of family and carers, also carried 100% agreement. There was 86% consensus agreement on ensuring that the healthcare professionals delivering aspects of the prehabilitation intervention should receive adequate training. Clarity of goal setting, adequate education of patients about the importance of prehabilitation and the reasons for any necessary behaviour change and education for families and carers achieved 86% agreement. There was 71% agreement on the need for coordination and regular meetings for the prehabilitation teams. Support and follow up using virtual methods like video calls or through an app, with an option for face-to-face appointments if needed or for those less familiar with technology also achieved 71% agreement.

### Outcomes

Thirty ideas were generated in response to the questions about outcomes to evaluate the effects and cost effectiveness of prehabilitation, with 12 achieving a consensus agreement of 70% or higher. The results of the individual positioning, thumbs up/down-voting and final individual ranking of the ideas and levels of agreement downloaded from GroupMap™ for prehabilitation outcomes are displayed in Supplementary file 3.

There was 100% agreement about the importance of patient stories and qualitative data. There was also 100% agreement on the importance of mental health outcomes to evaluate anxiety, stress and loss due to the disease’s effect on the patient’s life to evaluate prehabilitation. There was 86% agreement on the need to measure body composition at baseline, pre-surgery, at discharge and post-surgery. There was 86% agreement that grip strength should be measured as a useful gross overall strength outcome, with data time points suggested. There was also 86% agreement about the use of the 30-s sit to stand outcome measure as a predictor of strength and endurance and ease of use in a face-to-face or virtual settings. There was 71% agreement on the use of outcomes such as Cardiopulmonary Exercise Testing (CPET), the Six Minute Walk Test (6MWD) or incremental shuttle walk test (ISWT) as measures of functional capacity. There was 71% agreement on the need to monitor nutritional status, with weight and body mass index (*BMI*) suggested as outcome measures. There was 71% agreement on the need for monitoring fatigue, function and quality of life, given the high psychological impact of upper GI surgery, with longer term outcomes data points suggested at six and 12 months postoperatively. The EORTC–30 was suggested as the tool that should be used to monitor patient fatigue. There was 71% agreement on the need for monitoring cost-effectiveness of the prehabilitation intervention, including intensive care, high dependency and overall hospital length of stay and medication usage. The EQ-5D outcome, measured pre and postoperatively was also suggested as a measure of quality of life and cost effectiveness.

## Discussion

We found a high level of consensus to inform the development of prehabilitation interventions and guidelines. Core aspects that achieved consensus were (1) Prehabilitation for patients undergoing upper GI surgery should be multimodal and multidisciplinary and combine all three core aspects (exercise, nutrition and psychological support); (2) Should be individually tailored with patient opinions taken into account, as well as well-coordinated across multidisciplinary services; (3) Mental health and qualitative patient data should be monitored and healthcare professionals should be able to recognise when additional support may be required; (4) Peer support from survivors and involvement of family and carers is indicated.

Whilst there is limited evidence supporting multimodal approaches to prehabilitation in patients undergoing upper GI surgery [11], the consensus of our expert panel is supported by wider guidelines and evidence. Multimodal approaches are currently recommended by general cancer prehabilitation guidance [10], and there is a growing body of evidence to support the use of bimodal or multimodal approaches to improve functional capacity and postoperative outcomes (postoperative length of stay and severity of postoperative complications) in colorectal surgical patient populations [24, 25].

There was consensus about the need for prehabilitation interventions to be tailored to the individual patient. For this to be successful, there was also 86% agreement for training for staff to prescribe, deliver, tailor and evaluate prehabilitation interventions for patients with cancer. The panel recommended that the training should develop the skills needed to agree treatment goals, as well as the ability to support behaviour change conversations with patients. In addition, training should ensure any exercise components follow SAID principles (specific adaptation to imposed demands) in order increase intensity to facilitate progression, or reduce intensity due to treatment related side effects [26–28].

To guide prescription and monitoring, there was consensus that assessment of fitness and functional capacity was required. A wide variety of outcomes were suggested including both maximal and submaximal tests. Costs and training associated with assessments, as well as the ability and implications of supporting face-to-face or virtual settings were debated and due to this, flexibility in approaches was recommended.

There was also consensus on the need for flexibility around individual appointments and follow-up care to ensure accessibility and equity. There is growing evidence regarding the effective use of digital technologies to support or supervise home-based prehabilitation [29]. However, the results from this study highlight the complex nature of prehabilitation interventions. More work is needed in this area, particularly from a multimodal perspective that requires coordination of interventions for patients undergoing upper GI surgery. Furthermore, the need to involve family and carers and the importance of peer support (at a similar stage in their treatment journey) was emphasised by our stakeholder panel. There is evidence to support the use of peer support in some cancer populations [30]. There is also research demonstrating benefits but also acknowledging challenges associated with participation in cancer support groups given the unpredictable nature of cancer disease progression [31]. Many of the published reviews highlight the limited availability of research in this area and recommend more quality research evaluating the effectiveness of peer support on psychological outcomes to guide future interventions for patients diagnosed with cancer [31–33].

Whilst there was a perception that prehabilitation often focused on exercise content, there was consensus on the importance for individualised dietetic support, given the profound and long-term implications on the digestive system of patients undergoing upper GI surgery. This was further discussed in the consensus group meeting, and the patient advocate emphasised the importance of diet for individuals to be able to undertake exercise and to feel well enough to carry out daily tasks. This is consistent with studies of patient experience following upper GI surgery that highlight the scale of the changes that patients are required to make regarding quantity and frequency of meals, particularly following oesophageal surgery [34–36].

Psychological care for people with cancer is considered one of the three core aspects of prehabilitation interventions alongside exercise and nutrition; however, what constitutes an appropriate and effective psychological intervention, and who is best placed to deliver these as part of prehabilitation interventions remains relatively unknown [10, 37, 38]. The stakeholder panel were clear that it was essential to consider mental health and quality of life and therefore training maybe required to interpret these outcomes and identify when additional support is needed. Certainly, awareness of the roles of other healthcare professionals as well as guidance about when targeted or specialist interventions may be more appropriate, was deemed important. Training in an interprofessional environment using methods like simulation-based education may facilitate improved communication and awareness within the multidisciplinary team as well as understanding the synergistic effects of the multimodal components [39–41].

### Strengths and limitations

When considering the findings of this consensus study, it is important to acknowledge that the target sample size was 15 to 30 participants, and although 27 expressions of interest were received, only eight participants attended the online meeting. This was primarily due to clinical priorities affecting availability. Most importantly this meant that nursing, medicine and psychology professionals were not represented. High dropout rates are common in consensus studies, where a specific meeting date is required, for example a recent study experienced a 39% dropout rate between initial recruitment and the nominal group study online meeting [42]. Setting a suitable date that was inclusive to all was challenging, and we would agree that to overcome this issue, over-recruitment of representative groups may aid balanced representation on the day. The small number of participants and limited multiprofessional representation may have impacted on the breadth of ideas generated, especially as prehabilitation services are not currently standardised, leading to varied stakeholder experiences.

Alternative consensus method approaches such as the Delphi technique may have helped to overcome these limitations. The use of multistage, self-completed questionnaires with individual feedback to determine consensus from a larger group of experts used as part of the Delphi technique negate the necessity for a single meeting. However, as this process is often repeated a number of times, it can be lengthy (several months) to conclude, rather than generating immediate results. It is also considered to be less inclusive when lay people or patient representatives are included in the panel due to the lack of facilitated group interaction which allow for discussion, clarification and explanations, which was considered to be a strength in this study [16, 43, 44].

When generalising the findings, it is important to consider that participants were almost all from the UK with a focus on the UK health system. Given the level of complexity across multiple systems, delivery of a prehabilitation service will depend on multiple factors that include local context, resources and population needs alongside the developing evidence base, to ensure equity of access and best patient outcomes. The results of the study provide consensus on components and mode of delivery of interventions to inform the clinical implementation of prehabilitation for patients undergoing upper GI surgery. The high level of agreement across multiple aspects of prehabilitation interventions indicate that high levels of disagreement would be required by additional participants for consensus not to have been achieved. In a recent review, Steward et al. [45] discuss the challenges of implementing prehabilitation in resource-limited systems without widening potential health inequalities. Workforce capacity and capability, socio-economic status, health literacy and digital exclusion are therefore important considerations to successful implementation of this promising complex intervention and reinforce the need for diverse stakeholder co-creation in future prehabilitation research.

## Conclusions

The consensus panel findings are consistent with developing evidence trends and directions, extrapolated from other areas of prehabilitation evidence. It has confirmed core aspects that should be incorporated into prehabilitation for patients undergoing upper GI surgery and identified important considerations regarding the training of rehabilitation professionals. Further research is required to evaluate the effectiveness and cost effectiveness and accessibility of such multimodal approaches in this population. However, these valuable insights will help to facilitate prehabilitation services that are patient-centred and better integrated across professions and healthcare systems.

## Supplementary Information

Below is the link to the electronic supplementary material.

Supplementary Material 1 (PDF 320 KB)

Supplementary Material 2 (PDF 766 KB)

Supplementary Material 3 (PDF 537 KB)

## Data Availability

No datasets were generated or analysed during the current study.
