# Supplementary material for: Priorities for prehabilitation for patients with upper gastrointestinal cancer: a nominal group consensus study
Source: Support Care Cancer. 2025 Aug 18;33(9):794. doi: 10.1007/s00520-025-09844-5 (PMC12361275; doi:10.1007/s00520-025-09844-5)
Supplement: Supplementary file 2 — Supplementary Material 2 (PDF 766 KB) [file 520_2025_9844_MOESM2_ESM.pdf]

Supplementary file 2

Facilitator Roles in the mNGT process

A team of four facilitators conducted the online mNGT meeting (RS, OG, JR, MW). They were all physiotherapy researchers. Training of the mNGT process and familiarisation with the GroupMap^{TM} software was provided prior to the online mNGT meeting. During the online meeting, the facilitators were physically located in close proximity to optimise experience for all involved. A single facilitator ran each of the three discussion groups (OG, JR, MW). One facilitator (MW) with previous experience and expertise with the mNGT process conducted the ranking processes during the meeting. The fourth facilitator (RS) was the process coordinator, with responsibility for leading the introduction and ice-breaker activities, delivery of the initial presentation, managing the group transitions, timekeeping and troubleshooting any technical or individual issues during the meeting.

Consensus study online meeting agenda

**9.00 – 13.00**	**Morning session**
**9.00 – 10.30**	• Meeting overview<br>• Introductions<br>• Scene setting presentation<br>• Initial questionnaire results
10.30 – 11.00	*Screen / Coffee break*
**11.00 – 13.00**	• Breakout room discussions & clarification of ideas
13.00 – 14.00	*Lunch Break*
**14.00 – 16.00**	**Afternoon session**
**14.00 – 15.30**	• Summary of preliminary prioritisation results for each topic<br>• Discussion of preliminary prioritisation<br>• Final voting and ranking
**15.00 – 16.00**	• Preliminary results & conclusion<br>• Meeting close

**Timed 30 min discussion**		**Facilitator 2<br>Topic 1 (OG)<br>GroupMap™ 2**	**Facilitator 3<br>Topic 2 (JR)<br>GroupMap™ 3**	**Facilitator 4<br>Topic 3 (MW)<br>GroupMap™ 4**	**Additional breakout room for troubleshooting any technical or individual issues<br>(Facilitator 1 - RS)**
**10.45 – 11.30**		**Group A**	**Group B**	**X**	
**11.30 – 12.15**		**Group B**	**X**	**Group A**	
**12.15 – 13.00**		**X**	**Group A**	**Group B**	

Table to show group rotation format for the discussion & clarification stage (step 2) of the mNGT process
