# Supplementary material for: Priorities for prehabilitation for patients with upper gastrointestinal cancer: a nominal group consensus study
Source: Support Care Cancer. 2025 Aug 18;33(9):794. doi: 10.1007/s00520-025-09844-5 (PMC12361275; doi:10.1007/s00520-025-09844-5)
Supplement: Supplementary file 3 — Supplementary Material 3 (PDF 537 KB) [file 520_2025_9844_MOESM3_ESM.pdf]

Supplementary File 3 – Data downloaded from the GroupMap™ for the three topic areas

Table 1: Final Map for prehabilitation Interventions (n=28 ideas)

Ideas	Description	👍 (n)	👎 (n)	Comments	Position individually	Individually Rank	Final Vote	% agreement
**Multimodal Intervention – Physiotherapy, Dietetics and Psychology - GI Cancer Treatment**	Aiming to support the cancer cohort to manage their symptoms of cancer treatments i.e. muscle loss, fatigue, body image, social isolation etc. during their treatment phase	7	0		1	1	7	100
	Screening measure (i.e. Patient reported questionnaire) to establish if patients undergoing cancer treatment require additional support							
	For those needing support - assess each patient individually face to face in a clinic to establish impairments/difficulties and develop a plan to manage these during the treatment phase							
	Follow-ups in person (1:1 or group depending on patient needs) to review and progress							
	Telephone/virtual clinic for those living far away from hospital centres							
	Referral back to surgical team following intervention for consideration of surgery – easy referral back to prehab pathway if additional needs identified by surgeons							
**Individualised specialist dietetic support**		7	0		6	3	7	100
**Integration of peer support groups into multimodal prehabilitation interventions**		4	0		2	4	6	86
**Education sessions on what to expect post-op - a lot of my patients seem under-prepared on the extent of the surgery and how they will feel post-op, also on the expected progression of activity in hospital and on discharge**		3	0	Ensure peer support groups are included in any review of patient facing education materials	21	10	6	86
**Early access to MDT led course / programme of nutrition education / physical activity / psychological support / lifestyle coaching e.g. 6 week rolling programme of support with access to specialists for those who need more tailored advice e.g. specialist dietitians**		5	0		9	2	5	71
**Integration of smoking cessation and life-style changes through dedicated support**		8	0		17	6	5	71
**Exercise prescription tailored to the individual patient adhering to SAID principles**		5	0	Specific adaptation to imposed demands	7	8	5	71

				Programme needs to be individualised and progressively overloaded				
**Single point of contact person - coordinating role**		3	0		25	9	5	71
**Being able to offer a variety of modes for contact depending on what suits the patients**		7	0	Patient choice	12	11	4	57
**Tailored structured supervised gym-based exercise**		6	0	Including a warm up and cool down	13	12	4	57
				Multifactorial - including an assessment				
				Personalised medicine - specific exercises to target individual problems				
				Personalised medicine - including dose of exercise				
				Tailored based on the timing of surgery/where the patient is in the pathway				
				Incorporating evidence around sarcopenia				
				Whole Body Approach - SAID principles				
**Strengthening and Cardiovascular fitness exercises, group classes and individual programmes tailored to the interests and physical function of the patient e.g. home-based/chair exercises vs swimming/running plan for those more able and to suit their lifestyle, options for in-person and virtual classes, aligned with a coach/mentor/Clinical Nurse Specialist/Physio/ support person for motivation**		5	0		14	13	4	57
**Need to identify stakeholders and areas for improvement for patients. The difference between pre surgery and pre-treatment lies in increased symptom management for the pre-treatment prehabilitation**		3	0		23	20	4	57
**Peer support through group-based programmes - face to face or online**		4	0		15	21	4	57
**Best practice multimodal prehabilitation have a combination of prehabilitation exercises**		2	0		26	5	3	43

**(breathing and inspiratory muscle training), medication, psychological support and dietary changes which may also help the patient, all these together I believe will help the patient on the long run**								
**Joint clinic/same day appointment with MDT such as Physio, Nurse (cancer or ERAS for surgical), Dietitian, Occupational Therapy for wellbeing/sleep etc**		5	0		8	7	3	43
**Signposting to charities**		3	0	Understand charities that are available	3	14	3	43
**Structure home-based exercise sessions with pre-recorded demonstration/instruction**		3	0		16	16	3	43
**Digital exercise prescription, participation, lifestyle changes, sleep promotion and dietetic advice using a single app**		2	0	Tranerize™	18	22	3	43
**Collaboration with social support services**		2	0		4	15	2	29
**Specific diabetes management**		2	0		5	17	2	29
**Support ongoing prior to surgery, ideally face to face locally.**		3	0		19	18	2	29
**Follow up via video calls could be an option, may help those who are feeling a burden due to large amount of hospital appointments.**		4	0	Travel burden in addition to appointment burden	10	19	2	29
**High intensity interval training**		2	2	Exercise for tumour regression	28	23	2	29
**Inclusion of inspiratory muscle training into the pre-op regime**		0	2		22	27	2	29
**Walking / Cycling physical activity programmes**		1	0		20	24	1	14
**After the surgery, most of what have been done presurgery can continue but with some modifications depending on the patient condition. Some exercises might need modifications and to be started at lower levels then progressed gradually.**		1	2		24	26	1	14
**Exercise on prescription through other community providers**		1	2		11	25	0	0
**Pre-op teaching of Deep Breathing Exercises, supported coughing and logroll out of bed technique**		1	2	This could be grouped with education of what will happen postop	27	28	0	0

Table 2: Final Map for clinical implementation and behaviour change (n= 31)

Ideas	Description	👍 (n)	👎 (n)	Comments	Position individually	Individually Rank	Final Vote	% agreement
**Tailored to the individual**		8	0		5	1	7	100
**Ensure the patient opinion is valued i.e. what do they want to achieve out of prehab - Adherence**		9	0		6	2	7	100
**Peer support - hearing from those who have gone through the programme previously**		4	0		1	3	7	100
**Involvement of family or carers**		8	0		8	6	7	100
**Agreed level of training for anyone delivering aspects of programme/intervention appropriate level of staff member (similar to McMillian competency)**		2	0		3	4	6	86
**Goal setting**		6	0		15	5	6	86
**Helping people to understand why each component is suggested**		6	0		11	7	6	86
**Adequate education to patients of prehab importance and reasons for each change - Adherence**		6	0		18	8	6	86
**Patients' adherence could be improved by patients and family education, support groups, groups sessions. Along with the continuous support from health professions in different stages of the patient journey.**		3	0		21	15	6	86
**Monthly / Quarterly meeting for prehab team leads - Coordination**	Allows sharing of success/failure/improvement between services	7	0		13	12	5	71
	Comparison of service plans to see what works and what doesn't							
	Platform for continuous improvement within the NHS and beyond							
	Opportunity for research collaboration between services and organisations							
**Support for patients could be virtual video calls and via an app, but with an option for face to face for those unable to use the technology**		4	0		19	16	5	71
**Patients should have easy access and information about the different services. Good communication practices should be implemented among healthcare professionals involved. MDT meetings would be ideal. Common goals should be agreed. Involvement of family or carers should be encouraged to support adherence**		3	0		25	17	4	57
**Monitoring via method appropriate for the individual**		5	0		12	19	4	57
**Good communication from clinicians**		4	0		20	9	3	43
**Education for clinicians/MDT**		4	0		24	10	3	43
**Access to appropriate information**		2	0		4	11	3	43
**Weekly MDT meeting amongst all members of the Prehabilitation team - Coordination**		2	0		23	14	3	43

**Considering the long-term - e.g. the change of behaviour over a long period of time (what is the exit strategy?)**		2	0		2	18	3	43
**Strong links with community setting**		4	0		10	24	3	43
**Recognizing the impact on significant others**		3	0		7	13	2	29
**Independent prehabilitation service administration team - Coordination**	Coordinate referrals from surgical teams to the prehabilitation pathway	3	0		22	20	2	29
	Book and schedule appointments for patients in both face-to-face clinics and in follow up appointments (Face to face or virtual) allowing clinical teams to spend more clinical time with patients							
	Facilitate referral back to surgical teams following intervention from the prehabilitation pathway							
	Monitor patient sessions to flag when referral back to surgical team is required							
	Can monitor patient adherence levels from attendance logs taken by clinicians in all settings i.e. Face-to-Face clinic, group exercise classes, telephone/virtual follow ups etc.							
**Involve local authority service providers.**		4	0		9	21	2	29
**MDT clinics aligned with dieticians, psychological support and an anaesthesia assessment perhaps.**		3	0		31	23	2	29
**In the long run, conducting a guide that will help and guide health professions to deliver the best/updated programme to the patients in different stages. However, in the meanwhile education healthcare professions by online courses and conferences in hospitals that provide the service could be a good way to start.**		2	0		26	26	2	29
**Adherence - align with a mentor/coach/support person, group classes, regular re-assessment and progression of the programme to build motivation,**		1	0		28	28	2	29
**An app design with reminders to complete physical activity and also to rest/stretch and recover, reminders for hydration and sleep**		2	0		16	22	1	14
**Perhaps linked to a smart-watch or device that can record the activity to minimise "extra" tasks that patients have to do**		0	0		29	27	1	14
**Sport England and CIMSPA (Chartered Institute for the Management of Sport and Physical Activity) advice / support.**		2	0		17	30	1	14
**Potential for mobile application to monitor patient adherence**		1	0		14	25	0	0
**Compulsory?? programme of education sessions should be deliverable to all people requiring prehab. It could be online / face to face or hybrid to allow those from large geographical areas to all undertake the same program.**		2	1		27	29	0	0
**One prehabilitation service where different types of surgical patients could be referred, with some specialist input accessible for those that need it**		0	0		30	31	0	0

Table 3: Final Map for pre and postoperative outcomes (n= 30)

Ideas	Description	👍 (n)	👎 (n)	Comments	Position individually	Individually Rank	Final Vote	% agreement
**Patient stories - qualitative data**		7	0		2	5	7	100
**Mental Health - Anxiety and stress. loss. Due to the disease effect on patient life physically and mentally.**		7	0		12	8	7	100
**Body Composition at baseline, pre-surgery, discharge post-surgery with/without rehabilitation - (bio-impedance), (Ultrasound), MRI possibilities**		4	0		11	2	6	86
**Grip Strength - easy to do, quick, gross overall strength score. Best of 3 in dominant hand - Baseline, 6-8weekly, pre-surgery, post-surgery, post-NACT**		7	0		10	4	6	86
**30 sec sit to stand - could be done F2F/video call if needed. Easy to do, good overall gross strength and adds in an element of endurance**		6	0		22	9	6	86
**CPET, 6MWD or ISWT, STS test for those less able**	6MWD useful for demonstrating intensity to patients	6	0		1	1	5	71
**Nutritional status - well nourished**		3	0	Achieving weight control during prehab, treatment and post-surgery.	6	7	5	71
**Weight and BMI**		4	0		7	10	5	71
**Fatigue (EORTC-QLQ-30)**		5	0		16	11	5	71
**Function and QoL - it is a massive psychological impact having a large surgery like this, so it would be interesting to compare pre-op vs post-op vs 6-12m QoL**		2	0		24	16	5	71
**Cost- effectiveness - Length of stay (ICU, HDU, Hospital), medication usage, EQ-5D**		6	0		30	17	5	71
**EQ-5D pre & post op, rough measure of quality of life**		4	0		23	23	5	71
**Outcomes to Prescribe and Monitor Prehabilitation Interventions at baseline and before Surgery**	Weight + Height (BMI)	5	0		15	6	4	57
	Body Composition (Bioelectrical Impedance Analysis)							
	Calf Circumference (cm)							
	Hand Grip Strength (Hand grip dynamometer – Kg)							
	Cardio-Pulmonary Exercise Testing (CPET)<br>-Anaerobic threshold (AT)<br>-Heart Rate Recovery<br>-Work Done (Watts) - Oxygen Saturations (SpO_2) during Exercise (%)							
	6MWD (exercise tolerance)							

	Duke Activity Status Index – functional capacity							
	Maximal Inspiratory Capacity (cmH2O)							
	ACS-NSQIP SRC (American College of Surgeons) - estimates the chance of an unfavourable outcome (such as a complication or death) after surgery							
**Frailty score – Clinical Frailty Score (CFS)**		1	0		4	15	4	57
**Blood Pressure, heart rate responses**	Great pressure product	3	0		9	20	4	57
**Calf Circumference (cm) - Baseline, 6-8weekly, pre-surgery, post-surgery, post-NACT**		4	0		19	3	3	43
**SARC-F (sarcopenia) or DASI baseline, pre and postoperatively (6 week discharge)**		3	0		21	12	3	43
**Self-efficacy or confidence to cope**		1	0		17	22	3	43
**QoL should be measured, together with costs, side effects, complications and costs. Triaging, together with a discussion with the patient themselves, should indicate the type of prescription needed**		0	0		25	29	3	43
**Post-Operative Outcomes to Evaluate Prehabilitation**	Length of Stay with/without Prehabilitation (days)<br>Length of Stay on ICU / HDU with/without Prehabilitation (days)	2	0		28	13	2	29
	Body Composition at discharge post-surgery with/without prehabilitation							
	DASI at discharge with/without prehabilitation (discharge, 6 months post, 12 months post)							
	Quality of Life Questionnaire (e.g. EQ-5D-5L)							
**Functional Capacity measurement**		1	0		8	14	2	29
**Participatory measures - work, family,**		2	0		20	18	2	29
**Physical Activity e.g. General Physical Activity Quality - GPAQ**		2	0		14	19	2	29
**ACS NSQIP SRC- Baseline risk score to decide on invasive treatment or not - will prehab change the risk**		1	0		18	25	2	29
**E Notes / Diaries of Patient**	Capacity to exercise and completion?	2	0		26	21	1	14
**ARISCAT score for PPCs**		2	0		29	24	1	14
**After the surgery the same presurgery outcomes along with postoperative complications, length of stay in hospital. Also due to the disease effect on patient life and considering the high comorbidity associated following the surgery.**		1	0		27	26	1	14
**Number of healthcare usages – Emergency Department visits, Specialist nurse appointments, GP**		2	0		5	27	1	14
**Falls - FRAT?**		0	0		3	28	1	14
**WEMWBS / HADs type questionnaires**		1	0		13	30	1	14

Abbreviations Key: 6MWD = six minute walk test/distance; ACS-NSQIP SRC = The American College of Surgeons National Surgical Quality Improvement Program surgical risk calculator; ARISCAT = Assess Respiratory Risk in Surgical Patients in Catalonia; AT = Anaerobic Threshold; BMI = Body Mass Index; CFS = Clinical Frailty Score; CIMSPA = Chartered Institute for the Management of Sport and Physical Activity; CPET = Cardiopulmonary Exercise Testing; DASI = Duke Activity Status Index; EORTC QLQ-30 = European Organisation for Research and Treatment of Cancer 30-item; EQ-5D-5L = 5 level EQ-5D version measuring health related quality of life; ERAS = Enhanced Recovery After Surgery; F2F = face to face; GI = Gastrointestinal; FRAT = Falls Risk Assessment Tool; HADS = Hospital Anxiety and Depression Scale; GP = General Practitioner; GPAQ = General Physical Activity Questionnaire; HDU = High Dependency Unit; ICU = Intensive Care Unit; ISWT = Incremental Shuttle Walk Test; MDT = Multidisciplinary Team; NACT = Neoadjuvant Chemotherapy; QoL = Quality of Life; SAID principles = Specific Adaptations to Imposed Demands; SARC-F = A Simple Questionnaire to rapidly diagnose Sarcopenia; SpO_2 = Oxygen Saturation; STS test = sit to stand test; WEMWBS = Warwick-Edinburgh Mental Wellbeing Scale
